# Patient and provider barriers, facilitators, and implementation preferences of intimate partner violence perpetration screening

**DOI:** 10.1186/s12913-020-05595-7

**Published:** 2020-08-13

**Authors:** Galina A. Portnoy, Richard Colon, Georgina M. Gross, Lynette J. Adams, Lori A. Bastian, Katherine M. Iverson

**Affiliations:** 1 VA Connecticut Healthcare System, VA PRIME Center, 950 Campbell Ave, West Haven, CT 06516 USA; 2 Department of Psychiatry, Yale School of Medicine, 333 Cedar St, New Haven, CT 06511 USA; 3 Department of Internal Medicine, Yale School of Medicine, New Haven, CT USA; 4 Women’s Health Sciences Division, National Center for PTSD, VA Boston Healthcare System, Boston, MA USA; 5 Department of Psychiatry, Boston University School of Medicine, Boston, MA USA

**Keywords:** Intimate partner violence, Violence perpetration, Screening, Patient preferences, Provider preferences, Healthcare needs, Healthcare response

## Abstract

**Background:**

The substantial prevalence and consequences of intimate partner violence (IPV) underscore the need for effective healthcare response in the way of screening and follow up care.

Despite growing evidence regarding perspectives on healthcare-based screening for IPV *experiences* (i.e., victimization), there is an extremely limited evidence-base to inform practice and policy for detecting IPV *use* (i.e., perpetration). This study identified barriers, facilitators, and implementation preferences among United States (US) Veterans Health Administration (VHA) patients and providers for IPV use screening.

**Methods:**

We conducted qualitative interviews with patients enrolled in VHA healthcare (*N* = 10) and focus groups with VHA providers across professional disciplines (*N* = 29). Data was analyzed using thematic and content analyses.

**Results:**

Qualitative analysis revealed convergence between patients’ and providers’ beliefs regarding key factors for IPV use screening, including the importance of a strong rapport, clear and comprehensive processes and procedures, universal implementation of screening, and a self-report screening tool that assesses for both IPV use and experiences concurrently.

**Conclusions:**

Findings provide foundational information regarding patient and provider barriers, facilitators, and preferences for IPV use screening that can inform clinical practice and next steps in this important but understudied aspect of healthcare.

## Background

Intimate partner violence (IPV) refers to physical, sexual, and psychological aggression between current or former partners [1]. Those who experience IPV are at increased risk for numerous physical and mental health conditions, including cardiovascular, gastrointestinal, and respiratory problems, chronic pain, gynecological disorders, posttraumatic stress, anxiety, depression, substance use, eating disorders, and suicide [2–7]. In addition to adverse physical and mental health consequences for those who experience relationship violence, IPV presents a significant public health burden that extends to families, including children and the healthcare system as a whole. IPV increases the risk of housing instability and homelessness [8] and children who are exposed to IPV are at increased risk of emotional, behavioral, physical, social, and academic problems [9]. Additionally, approximately $10.4 billion is spent in the U.S. and £1.4 billion in the U.K. in healthcare costs annually associated with IPV ([10, 11], respectively).

The substantial consequences of IPV underscore the need for effective healthcare response in the way of screening and follow up care [12, 13]. Although several key agencies, including the U.S. Preventive Services Task Force and Veterans Health Administration (VHA), have issued recommendations for routine screening for IPV experience (i.e., *victimization)* in healthcare settings [14, 15], there is limited research to inform screening practice and policy for identifying individuals who use violence in relationships (i.e., *perpetration*). Screening individuals for IPV experiences is essential to providing the necessary support and follow up care. Yet, given the scope and far-reaching consequences of IPV, secondary and tertiary prevention by way of screening for IPV experiences alone is insufficient. Prevention of IPV requires the detection of IPV use and referral to follow up care focused on ending IPV and preventing future acts of violence. In response to the major health concern that IPV poses in the lives of Veterans, the VHA has adopted a holistic, person-centered, psychosocial rehabilitation framework to inform IPV response [16] Because shifting the language used to refer to IPV can help to decrease stigma and barriers to care, VHA recommends the use of person-centered language including “Veteran who uses IPV” instead of “batterer, abuser, or perpetrator” and “Veteran who experiences IPV” instead of “victim or survivor”. Labels, such as “perpetrator” and “abuser” can also serve as barriers to honest reporting of IPV behavior and/or accessing treatment.

Although effective treatment for Veterans who use IPV is becoming more readily available in VHA [17], a gap in knowledge regarding appropriate screening practices remains. Some screening instruments for the detection of IPV use [18, 19] and general violence [20, 21] have been developed for healthcare settings, but there is little research to guide their use in routine care. Only a handful of studies have been conducted exploring IPV use screening in healthcare settings in the U.S. [18, 22, 23] and internationally [24], and even less in the VHA [25], the largest integrated healthcare system in the United States (US).

Foundational research identifying VHA patient and provider perspectives on addressing IPV use is essential because of its high prevalence among Veterans. Estimates of IPV use among Veteran men ranges across studies from 28 to 60% [26, 27]. Fewer studies have examined IPV use among women Veterans, yet in those that have, rates are substantial (e.g., 17% past-year IPV [19];). Although the rate of IPV use among Veterans varies significantly, even the lowest estimates indicate that at least 1 in 6 Veterans have engaged in IPV and those with comorbid psychopathology, such as posttraumatic stress disorder (PTSD) and/or substance use disorder, are at increased risk of using violence [20, 28]. Despite the high rates of IPV use among Veterans, best practices for routine IPV use screening at VHA and broadly in non-Veteran healthcare settings are lacking. Additionally, scarce research exists to inform IPV use screening implementation and little is known about provider and patient preferences for IPV use screening. Some challenges to screening male patients for IPV use in healthcare settings have been presented, including providers’ discomfort assessing risk and lethality, concerns discerning their legal obligations regarding duty to warn, and worries about misidentifying the patient as a victim [22], although the extent to which of these challenges are generalizable to VHA is unknown. In order to inform the development of IPV use detection efforts in VHA, knowledge of Veterans’ and providers’ experiences, behaviors, and preferences for IPV screening is critical, and such findings could have implications for detecting IPV use in other healthcare systems. As such, qualitative research with these key stakeholders is a crucial next step.

The development and implementation of screening and counseling practices to address IPV use requires a nuanced understanding the needs and preferences of the target populations [29]. Understanding the perspectives of those who will be involved in or affected by implementation of an intervention is critical to ensuring that interventions address problems recognized as high priority and use approaches that are feasible and acceptable in real-world care settings [34]. Prior to the implementation of IPV screening programs in VHA to identify women who experience IPV, women VHA patients and providers were asked their preferences and perspectives for IPV screening and response procedures [6, 30–32]. Similarly, VHA patients and providers can assist in pre-implementation efforts for IPV use screening, such as identifying potential challenges and concerns, offering recommendations for successful implementation and uptake of screening, and elucidating factors that may enable effective screening. The aim of this study was to explore VHA patients’ and providers’ perspectives on IPV use screening, with a focus on barriers, facilitators, and implementation preferences.

## Methods

### Setting, participants, and recruitment

This qualitative research study was conducted at a large US VHA medical center located in the Northeast. Analyses are based on in-depth semi-structured interviews with Veterans (*N* = 10) and focus group discussions (FGDs) with providers (*N* = 29; 6 focus groups). We used purposeful sampling strategies to recruit Veteran and provider participants [33]. The research protocol was approved by the local VHA Institutional Review Board.

#### Qualitative interviews with veterans

Veteran participants responded to recruitment fliers posted throughout the VHA medical center and scheduled for interviews following a phone eligibility screening conducted by the lead author. Veterans provided written informed consent and received $50 compensation for their participation. Both interview guides (Veteran and provider) were developed for the purpose of this study and are provided as Additional File 1. The Veteran interview guide was structured to elicit Veterans’ past experiences and/or anticipated reactions to being asked about IPV use screening, facilitators and barriers to reporting IPV use behavior, and preferences for IPV use screening. For example, Veterans were asked: “How would you feel or react if a VA healthcare provider asked you about your violence or aggression towards a partner?” “What might get in the way of talking with your healthcare provider about these topics?” “What would make it easier to talk with your healthcare provider about these topics?” Interviews lasted 50 min on average. Four Veteran participants identified as Black or African American, 4 as White, 1 as Hispanic; and 1 declined to answer. Veterans’ mean age was 55.3 years (range = 30–69 years). The majority of Veteran participants were men (1 woman, 9 men). We continued recruitment until we reached thematic saturation [35].

#### Focus group discussions with providers

To recruit providers for FGDs, the lead author attended staff meetings in primary care and mental health clinics, described the study, and requested participation. Interested providers were contacted and divided into six FGDs based on their availability. We received a waiver of written informed consent for provider FGDs and providers were not compensated for their participation. The focus group interview guide was structured to elicit providers’ past experiences and/or anticipated reactions to screening Veterans for IPV use, and facilitators, barriers, and preferences for IPV use screening. For example, providers were asked: “Have you ever asked patients whether they use violence or aggression towards a relationship partner (such as physical violence, sexual violence, psychological or emotional violence, or stalking)? What are your reasons for asking or not asking?” “In what circumstances do you ask?” “What gets in the way of asking patients about their use of violence and aggression in relationships?” FGDs lasted 46 min on average. Provider participants were mostly women (21 women, 8 men) and included 12 psychologists, seven social workers, four psychiatrists, three nurse practitioners, two physician/primary care providers (MD PCPs), and one marriage and family therapist. On average, FGDs contained 5 participants (ranging from 4 to 7 participants).

### Data collection and analysis

All interviews and FGDs were conducted face-to-face in a private room at the local VHA facility by the first author, an experienced qualitative researcher. An assistant moderator took notes on non-verbal activity (e.g., body language illustrating consensus/difference), helped minimize distractions, and participated in field note generation following FGDs in order to identify initial reactions, reflections, and observations. Throughout interviews and FGDs, the interviewer performed member checking by paraphrasing and summarizing content to clarify intent. Data collected continued until we reached saturation. To protect confidentiality, Veterans were assigned a study identification number (which was later converted into a pseudonym) and providers were asked to come up with a pseudonym to be used during FGDs. Interviews and FGDs were digitally recorded, professionally transcribed verbatim, stop-checked for accuracy, and de-identified.

The qualitative research team included seven interdisciplinary members with varied IPV content knowledge and methodological expertise, led by the study first author, a female research psychologist with expertise in IPV. In Microsoft Excel, we conducted qualitative analysis of the transcribed interviews using a combined inductive and deductive analytic approach combining a priori thematic analysis and content analysis [36, 37]. Using a team-based, consensus approach, we identified the following key domains based on the interview guides: factors that interfere with IPV use screening (i.e., barriers); factors that enable IPV use screening (i.e., facilitators), and implementation preferences. These domains were used to develop a transcript summary template [38].

The research team was split into pairs to conduct line-by-line coding and transcript summarizing. Each coder independently coded and summarized the same transcript attending to targeted, deductive domains previously identified, new inductive codes that emerged, and exemplar quotes. Coding pairs convened to discuss and refine code definitions, fit of codes to transcript summary template, and reach consensus across codes and summaries. The full research team met regularly to align coding and summarizing, synthesize codes into themes, and resolve discrepancies through discussion until consensus was reached. Finally, the research team collaboratively and iteratively reviewed, discussed, and sorted the codes to refine themes and sub-themes and select exemplar quotes. Lead author presented qualitative findings to a subsample of providers involved in FGDs for their questions and feedback.

## Results

Inductive analysis resulted in the identification of 12 sub-themes. We categorized related sub-themes by the overarching domains: barriers, facilitators, and implementation preferences. As described in detail below, patient-specific barriers and facilitators that may interfere with or aid in IPV use screening included rapport, reactions to screening, and clear and comprehensive processes and consequences. Provider-specific barriers and facilitators included preparedness to conduct IPV use screening, logistical and procedural factors, providers’ scope of practice and role, providers’ own reactions to IPV and expectations for screening, and the salience of IPV to patients’ clinical presentation. In both patient and provider samples, two sub-themes emerged regarding the method for screening and setting preferences for IPV use screening implementation. Table 1 displays summarized findings. We present the overarching themes and sub-themes alongside representative quotations using pseudonyms for patient participants and professional discipline for provider participants.

**Table 1:** Summary of findings

Theme	Definition	Subthemes
Patient barriers and facilitators	Patient-specific factors that interfere with or aide in IPV use screening	− Rapport
		− Reactions to screening
		− Clear and comprehensive processes and consequences of screening
Patient implementation preferences	Patient identified preferences for IPV use screening implementation	− Methods for screening implementation
		− Setting of screening implementation
Provider barriers and facilitators	Provider-specific factors that interfere with or aide in IPV use screening	− Preparedness
		− Logistical and procedural factors
		− Scope of practice and provider’s role
		− Providers’ reactions to IPV and expectations for screening
		− Salience of IPV to patients’ clinical presentation
Provider implementation preferences	Provider identified preferences for IPV use screening implementation	− Methods for screening implementation
		− Setting of screening implementation

### Patient barriers and facilitators

#### Rapport

Patients consistently noted that rapport played a key role in their IPV use screening experiences as did anticipated reactions from providers regarding disclosure of IPV behavior. Poor rapport contributed to an unwillingness to discuss and disclose IPV use to providers. Patients attributed poor rapport to having a negative (or no) previous relationship with the screening provider, and therefore not having a trusting relationship from which to be open and vulnerable with the provider or understand why the provider is asking about IPV. Patients also stated that if it felt as though the provider was ‘just checking something off a list’ or they had negative perceptions of the provider, this affected rapport. For example, Alex commented, “When you feel like you’re just being pushed through, like on a conveyor belt, you’re not as readily going to open up.” Similarly, Mallory noted, “Some [providers] I don’t trust… if they asked me those questions, I would shut down.”

Alternatively, strong rapport emerged as a salient factor that enabled IPV use screening. Patients spoke to the importance of trusting the screening provider, having an established relationship with them, and believing that they were administering the screening to support the patient and facilitate their care. Overwhelmingly, patients described the strength of rapport and aspects of the provider’s style that increased acceptability of screening and likelihood of disclosure, including being non-judgmental, unbiased, normalizing, caring, and genuine. Corey indicated, “This is hard core, you’re not sparing no punches. Give me a chance to get used to you first before you ask me something like that.” During Morgan’s interview, they similarly noted, “If I felt like they genuinely cared, even though it might be a routine thing, if I felt that I had established some rapport, I might be more apt to answer truthfully”.

#### Reactions to screening

Patients were asked about past experiences in which providers assessed for IPV use. The majority of patients reported that they had not been asked about IPV use. Those who had not had experiences with screening were asked to consider how they may respond and react to a provider directly inquiring about IPV use. Some patients expressed ambivalence about how they would feel about IPV use screening, including having some negative reactions. These patients stated that they might feel offended, caught off guard, or targeted, while others noted that IPV use screening could be triggering to them or that they would experience shame, stigma, and concerns regarding judgement from the provider. For example, Mallory stated, “I would get angry that it seems like they [providers] were accusing me, that they were like trying to get something out of me, like I’m not telling them the whole truth.” Additionally, Quinn spoke to the emotional toll screening for IPV use could take, “When I relive the experiences, past experiences, it makes it difficult sometimes because I feel them and I remember them and that’s what makes it difficult.”

However, anticipating experiencing a positive reaction from providers and openness to screening served as facilitators. In fact, most patients reported that they would not feel defensive or offended by IPV use screening and/or that they would answer such questions honestly as long as they felt comfortable with the screening provider. For example, although Quinn (quoted above) worried about being triggered by screening, later in the interview the Veteran described that they anticipated disclosing IPV behavior honestly and would perceive the provider as helpful:

*I understand it’s to help me, so I don’t have a problem to open up… I understand that it’s just to try to help me deal with things better… I can be honest about it because I’m trying to be a better person and try to just get through these things I’m dealing with. (Quinn)*

#### Clear and comprehensives processes and consequences of screening

Patients expressed concerns regarding lack of clarity regarding clinical care and documentation following IPV use screening, including potential consequences that they could face following disclosure of IPV use. Fear of consequences included legal concerns due to providers potential mandated reporting requirements. Privacy and confidentiality concerns about how IPV use would be documented in their electronic medical records was also a key issue for patients that would interfere with patients’ willingness to be forthcoming about their behavior. In addition, patients indicated that they would be reluctant to report IPV use unless they believed there would be a helpful follow-up discussion with the provider, including referrals to related services or future incorporation of the issue into treatment.

*If I did have that problem, I would like to know that I'm not going to go to prison just because I’m admitting to something I did… the first thing is you’re going to take away my guns, the next thing you’re going to lock me up, next it’ll go on my record now. Now I can’t get a job. No one hires domestic violence, no one. It’s a very touchy subject because Veterans are very protective of their freedom and their liberties. (Angel)*

*Sometimes people ask you something and then they don’t do anything about it, you know? If I were to admit that [IPV use] was going on, and then they didn’t do anything about it, I think I would be a little upset. What would be upsetting about it is the fact that you’ve kind of opened up a wound and then you don’t close it back up. I poured my heart out to you, or I talked about some intimate kinds of issues, and you don’t refer me to someone, that would be upsetting to me. (Morgan)*

Having comprehensive and clear procedures for IPV use screening seemed to enable patients’ disclosures. Specifically, when patients believed that providers would follow up on positive screens and provide referral options, they indicated they would be more likely to disclose IPV. For example, Sam stated, “I would need to know if there’s any kind of follow up care. If you’re asking me those questions then I mean, what’s the point in asking me if there isn’t any kind of help so to speak?” Additionally, patients overwhelmingly spoke to the importance of being asked about IPV experiences as well as use. The ability to share “the full story” (i.e., assessment of both IPV experiences and use) during a comprehensive IPV screen was suggested as important for enabling the screening process and facilitating disclosure:

*I understand why the question would be asked, you know, just from the culture of the military in general. But I think for me, it would probably be helpful to not just be asked if I’ve been violent but have I also been a victim of violence because I feel like a lot of times the world that we live in immediately assumes that either the Veteran or the man is immediately the violent one and sometimes it could be the other way around. (Jordan)*

### Patient implementation preferences

#### Methods for screening implementation

There appeared to be some consistency regarding the preferred methods for IPV use screening procedures. Patients noted the benefits of completing an IPV use self-report screener prior to their healthcare appointment (along with other relevant health and psychosocial screeners), with the provider following up on the questionnaire during the visit. For example, Jordan stated, “For me it’s a lot easier to fill out a form and then be asked questions based on that form rather than that face-to-face conversation with no information being provided already.” Additionally, patients recommended self-report tools contain a rationale and introduction to the IPV use screening items and preferred for IPV use screening to be routine so that they do not feel targeted, for example, as part of an intake process or at an annual primary care visit:

*If a provider said this is a routine question that we ask everyone, then I think I would feel okay with that... that would be important to me because I wouldn’t want to feel that I was being targeted for some reason...I don’t think I would feel offended as long as this was a routine question that’s being asked of everyone. (Morgan)*

#### Setting of screening implementation

Although consensus emerged regarding the method for IPV use screening, patients did not agree on the setting in which the screening should occur. Some Veterans reported being comfortable and willing to answer IPV use questions in primary care when it was done as part of routine screening for psychosocial health issues. However, others, like Avery, expressed strong unwillingness to answer IPV use questions in primary care and preferred that the screening take place only in mental health settings, “You know I’m going there for my physical health…So I wouldn’t feel comfortable with that because it’s not the type of setting for that to me…. [I’d be] very, very, very much offended.” Those who preferred screening to be done in the mental health context did so because they expressed a stronger rapport with their mental health provider and/or because they believed that IPV was outside the scope of primary care. Alternatively, patients who expressed preference for IPV use screening in primary care believed that this was the appropriate setting because primary care reaches more patients.

*I think that I would be okay talking to either, but I feel like a lot of people have trouble getting to a mental health provider so I feel like the primary care would be the better start. (Jordan)*

*There’s a lot of Veterans that don’t see mental health, that hardly ever go to primary care. They go in once a year. But if you give this to them and they see this, maybe someone will have luck in the future that an action or a comment will trigger a memory to this and they can say okay, yeah, the VA asked me about that. Let me go to the VA. (Angel)*

### Provider barriers and facilitators

#### Preparedness

Providers’ perceptions of their preparedness to carry out tasks related to IPV use screening impacted their current screening practices and their willingness to screen. These tasks included feeling prepared and knowledgeable in 5 domains: (1) what questions to ask and how to ask them, (2) how to document IPV use in patients’ electronic medical records, (3) understanding IPV-related mandated reporting requirements, (4) knowing available referral options, and (5) how to optimally follow up on positive screens.

*I’m not really sure what I would do in the situation of a Veteran being the perpetrator. I guess it depends on what’s happening. I mean obviously I'm always keeping an eye on safety and whether or not they’re safe to reside in the community. But honestly, I don’t think I know. I know what to do if the person is the victim but not for the perpetrator. (Amanda, ER Social Worker)*

*When I have asked in the past, I feel like I’m not confident that I’m using the right terminology or the language that’s going elicit responses. If I’m going to assess for it, I want to be more confident that my assessment is going to be appropriate and get accurate information. (Mia, Psychologist)*

*I don’t know enough resources to give direction. With suicide I have that pretty readily available. When it comes to IPV, I don’t have those resources at my ready. (Thomas, Psychologist)*

#### Logistical and procedural factors

Related to factors that impact preparedness, many of the barriers and facilitators identified by providers were logistical and procedural, such as the set up of the clinic, clinic policies, services available for referral, and current supports in place to facilitate IPV use screening. Other specific logistical and procedural barriers included limited time in session, lack of an IPV diagnosis and diagnostic code for IPV, limited treatment options for referral, and IPV not being the presenting problem. Steve, a psychologist, noted, “We’re focused on DSM diagnoses. IPV is maybe part of PTSD, part of irritability, part of bipolar disorder? It’s not its own entity and we may not know specifically what the treatment would be for it.” Another mental health provider discussed the challenge of limited time in session, a concern shared by most providers across disciplines:

*I’m limited to my time and I just want to get in and talk about the things that they’re presenting for and if it’s not that [IPV] then I may not want to ask those questions. It can start to open up things that you don’t have the ability or the time to really get into. (Mariah, Social Worker)*

Conversely, when policies, logistics, and procedures supporting IPV use screening were clear and providers believed that their clinic and supervisor supported IPV use screening, these factors enabled screening. One example of clear procedures was providers’ desire to have an IPV use screening tool available. Multiple providers noted that such an instrument would help guide the screening process, provide a structure for referrals, and facilitate discussion of IPV.

*I think the other thing that I think would be helpful for me is really understanding VA’s policy or recommendations for it. I feel like I’ve never really received any guidance on that here and because the VA obviously emphasizes patient care and kind of really prioritizing that you obviously don’t want to do anything that’s going to really damage rapport with the patient where they don’t want to come back here for care. So really trying to understand their policy or recommendations for that would be helpful for me. (Charles, Psychologist)*

*I would like a clear-cut screening tool. I think that would help. What questions should we ask because we really haven’t had training in that area. Like, we work towards the victim so that’s what would be helpful to me is a screening tool. (Abby, Social Worker)*

#### Scope of practice and provider’s role

Providers’ perceptions of whether IPV (and specifically IPV use) fit within their scope of practice served as a major factor that either enabled or interfered with IPV use screening. Those who did not perceive IPV as within their scope of practice were unlikely to routinely screen for IPV use and did not believe it was their responsibility to incorporate screening into routine care. For example, Richard, a male psychologist, noted, “It’s the same way that I feel about pain – it’s pervasive and involved in so many areas, in the similar way that interpersonal violence is. It becomes this sort of thing that’s specialized but it’s not what I do.” Relatedly, when providers perceived IPV use as outside of their scope of practice, they expressed concerns regarding liability, especially in the context of low confidence regarding documentation and reporting requirements.

*The thing that I am most concerned about in this situation is liability. If I document all of this stuff and didn’t protect the victim, then something really bad happens and I’ve documented all of this, then does that mean that I’m responsible if they go and hurt someone? (Joan, Psychologist)*

#### Providers’ reactions to IPV and expectations for screening

Some providers expressed discomfort regarding IPV and avoidance of having conversations related to IPV with their patients. For example, Robin, a psychologist, explained, “When I ask it, I’m a little uncomfortable and then I’m not making it that easy to talk about.” Providers were also concerned that bringing up IPV would damage rapport and/or make their patients feel uncomfortable. Other providers expressed concerns regarding their own safety, worrying that initiating conversations about violence would put themselves at risk. Additionally, providers’ biases regarding patients who use IPV impeded their likelihood of screening. For example, when providers expressed beliefs that patients lack insight about their own behavior or that they would not tell the truth or will minimize, providers were less likely to inquire.

*I haven’t actually worked with a Veteran who has shared that he or she has committed IPV. If they mention anger and aggression issues, I think the barrier would be that I can sense them already becoming uncomfortable and I nip it in the bud. So for the sake of the rest of the session, I feel like it could be jeopardized at times. There are times I think I probably should have been more thorough and maybe I wasn’t. (Evan, Social Worker)*

*In two instances I can think of, one of the things that did float through my head is that is my safety in danger having this other person knowing that I am now involved in some possible legal ramifications that will directly affect them. (Dion, Nurse Practitioner)*

*I think that some providers just give up and think they’re not going to tell me anyway. Even though I’m sure of all those people that I’ve seen over the past 15 years I’ve been in the field, likely somebody has [perpetrated IPV]. There’s no way that it’s that small of a number. (Colleen, Psychologist)*

Alternatively, when providers believed that patients would be receptive and not offended by IPV use screening, they were much more likely to screen. For example, Andrew, a social worker, stated, “I think that people want to be asked, it’s a burden that people carry.” Additionally, many providers, like Steve, a psychologist, expressed being more comfortable to screen for IPV use when they believed that they already had a strong rapport with the patient, “If you have a good rapport with someone then you’re better able to get them to open up and feel comfortable.” Others noted that, for them, asking about IPV use is no different from asking about other sensitive topics:

*I don’t think it’s ever been like “I hate you for asking or angry that you’ve asked” because I also ask lots of other uncomfortable questions like sexual orientation and all sorts of things that I don’t think anyone’s caught off guard that I’m also asking other uncomfortable things. (Stacy, Psychologist)*

#### Salience of IPV to patient’s clinical presentation

Across disciplines, providers stated being much more likely to assess for IPV use directly when patients presented for care related to similar concerns or when ‘red flags’ came up in session. Dion, a nurse practitioner, stated, “It was something in their presentation that made me ask or something specific to what they said, but it’s not with everyone.” Providers, especially those with specialized training in behavioral health, described that they are prompted to assess for IPV use when a patient has a PTSD diagnosis or concerns related to relationship challenges, anger management, and substance use. For example, Danielle, a psychologist, stated, “In substance use outpatient treatment, when people will kind of imply that they’ve gotten so angry or been so intoxicated that something has been about to happen or has happened, I will ask more questions about it.” Finally, providers agreed that screening is easiest when patients bring up IPV or violence themselves:

*I have plenty of guys who come in and talk about violence, so I’ll ask them about that. I would frame it as, oh, so this is a problem that you’d like to work on? And try to move in that direction. (Alex, Psychiatrist)*

### Provider implementation preferences

#### Methods for screening implementation

Many similarities emerged between providers’ and patients’ preferences for the methods by which IPV screening should be implemented. For example, like patients, providers also preferred that screening be universal (e.g., routinely incorporated into practice rather than targeted), conducted using a screening instrument, and assess IPV use and experiences concurrently. A general internist discussed the benefit of universal screening, using a screening instrument:

*I think from an epidemiological public health perspective, you start with the screening. Asking the question gets the answer. You can show that screening instruments are good and that actually doing the screening and sending somebody to treatment actually makes a difference. (Ava, MD PCP)*

Additionally, Greg, a psychiatrist, noted what would be lost by only screening for IPV use without including experience (i.e., victimization) screening, “I think [screening for both] just gives you a fuller picture of what’s going on, like I don’t think I’d want to hear just one half, you know?” A suggested method for implementing IPV screening into routine care was to conduct IPV screening during risk assessment along with suicide and homicide screening or to integrate it as part of routine intake procedures for patients of all genders:

*In the last year we’ve started doing a standard assessment as part of our intake procedure. We do a questionnaire on both their use of violence and also their experience of receiving violence. That’s been very helpful in terms of having a structured way to answer questions. A benefit of asking everyone is making it more salient and somewhat more normalized and that we know this is happening, and it could be happening to anyone, anyone could be doing it. (Molly, Social Worker)*

#### Setting of screening implementation

Providers’ broad range of opinions regarding preferences for the setting in which IPV use screening should be implemented attests to the breadth of responsibilities, clinic policies, and provider needs across disciplines. Some providers conveyed a strong preference for IPV use screening to occur in primary care due to its broader reach, patients’ difficulty following through on referrals to mental health, and because a wide range of screenings are already conducted in primary care, making screening an expected part of the primary care culture and experience for both patients and providers. For example, Elle, a psychologist, noted, “We know that most people that probably need mental health care and are referred from primary care don’t come. So we’re going to be limited in who we catch just based on that.” Others believed primary care was an appropriate setting for screening because IPV use is not necessarily a mental health issue.

*I bristle against the idea of the responsibility falling [only] on mental health. There’s not a one-to-one correlation between this and mental health diagnoses. You know, kind of like we’re seeing with the nationwide debate on gun violence right now and everybody’s screaming mental health diagnoses. A lot of the people that are perpetrating gun violence are not necessarily people who have well-established mental health diagnoses. So I really believe that the screening should take place at a broader place where it can catch more Veterans who are potentially involved in this. (Tanya, Psychiatrist)*

*I suppose it’s whether you feel like everybody should be screened or whether you feel like they are at a higher risk and should be screened? If it’s everyone, then, I hate to say it, primary care is probably the answer. (Francis, MD PCP)*

Despite the strong rationale for screening in primary care, both primary care and mental health providers believed that mental health providers would be more effective at implementing IPV use screening given their training and focus of services. As stated by Robin, a psychologist, “It just seems like the top of what one should worry about and respond to in mental health. Suicide, loss of control, anger. I think that mental health, if there is a place to routinely ask, that’s the place.” Overall however, many primary care providers strongly disagreed that IPV use screening should occur in primary care and stated that it should instead be implemented in mental health clinics. These providers rationalized that a mental health setting was better equipped to “handle” a positive screen and that such a screening requires more attention and discussion than a checklist would provide:

*The volume in primary care is unbelievable, and you don’t know who’s coming in with what, so the volume is too much. We have to assess very quickly what this person is coming in for, are they safe, is this just a quick little transaction or is this a longer thing? It’s very difficult in primary care. (Catrina, Primary Care Social Worker)*

The discussion regarding implementation setting extended beyond mental health or primary care. Some providers, like Andrew, a social worker, believed that IPV use screening should be widely implemented in all settings or in multiple settings to enhance reach and decrease stigma: “Any point of contact. Like, to normalize it. If it becomes a standard question, it won’t be an abrupt or jarring experience for the Veteran to be screened.” Others suggested that screening be conducted in the settings in which a patient first engages in care:

*I think it’s got to be broad and I think it’s got to be everywhere. Many years ago, when we started the TBI screening it was whoever picked up the chart if that reminder was due that person did it. I think everybody’s got to be looking out. I think everybody’s got to be aware of it because otherwise we’re going to miss a lot. (Ashley, Nurse Practitioner)*

*It’s a really good idea to put it in place where we’re going to catch the largest number of Veterans, because certainly not every Veteran is in mental health… I also wonder about other entry points of care for people. I don’t know if there are other places here that are Veteran's first connection with the VA. (Jemma, Marriage and Family Therapist)*

## Discussion

This qualitative study is among the first to comprehensively examine both patient and provider perspectives on IPV use screening, and is the first to assess barriers, facilitators, and implementation preferences regarding IPV use screening in the VHA. This study found many areas of convergence between the preferences of patients and providers perceived facilitators and barriers to IPV use screening, as well as recommended methods and settings for implementation of screening. There was a lack of consensus regarding preferences for the settings in which IPV use screening should be implemented not only between patients and providers but within both groups as well. Given the rate of IPV within the Veteran patient population and the lack of screening currently conducted for this prevalent health and healthcare issue, these findings provide foundational knowledge for informing practice and policy for IPV detection and care in healthcare settings.

### Convergence of patient and provider feedback

Patients and providers agreed that one of the most important factors for successful IPV use screening was strong rapport. Patients stated that they need to feel connected to and trust their provider to honestly discuss IPV; this finding extends research highlighting ‘connectedness’ as a key enabler of disclosing IPV *experiences* [31]. In turn, providers expressed feeling uncomfortable asking questions about IPV use in the absence of a strong relationship with the patient or if they did not expect that the patient would respond positively. This finding parallels existing literature on barriers of screening for IPV experiences in VHA showing that a sensitive and empathic approach, ongoing relationship, and comfort with the provider are essential to women Veterans’ willingness to discuss IPV experiences [6, 30–32]. Similar observations have been documented among non-VHA providers and patients, in which a respectful and trusting relationship was shown to be important and associated with sensitive IPV screening and disclosure [39, 40].

Another facilitator that patients and providers agreed on was the importance of clear and comprehensive processes and procedures following IPV use screening. Patients believed that they would be more open about IPV use behavior if the consequences of disclosure (i.e., documentation in electronic medical records and possible mandated reporting requirements) were clear and referral services were available. Similarly, providers noted significant barriers to screening in the way of unclear procedural and systematic factors related to clinic policies, leadership support, and resources available for positive screens. Concerns related to the consequences of a positive screen are likely worsened by the varying legal requirements across states [22]. Findings from the present study extend the literature on IPV detection in VHA to include similar concerns regarding IPV use screening. Research with patients and providers prior to implementing screening for IPV experiences highlighted patient and provider concerns of regarding negative consequences of disclosure [30], logistical and educational barriers to screening [6, 32], and the importance of follow-up support, transparent documentation, and availability of resources [31]. Patient and provider concerns regarding next steps following IPV use disclosure are sensible given that only one empirically supported treatment for IPV use among Veterans exists to date, a trauma-informed group intervention for men [17]. More broadly outside of VHA, the evidence for IPV use treatment in healthcare settings is weak [41]. The development of additional evidence-based interventions for this population is crucial and the need for additional treatment options is reflected by patient and provider reluctance to engage in IPV screening without adequate follow up services.

Three method-related screening preferences were consistent between patients and providers. First, both groups agreed that there could be benefits to universal implementation of IPV use screening in order to reduce stigma and enhance reach. Second, both patients and providers spoke to the acceptability and appropriateness of implementing a self-report screening tool in routine care. Patients and providers desired a clinical tool for IPV use that patients could complete in the waiting room prior to their appointment. In fact, an IPV use self-report screening approach has shown success in prior research [18]. However, patients noted that a self-report screener would only be effective if the provider followed up on patient responses. Third, both patients providers agreed that the screening tool should inquire about both IPV use *and* IPV experiences, consistent with prior research highlighting the value of concurrent screening for IPV [22]. In our study, findings demonstrate that patients are more likely to disclose IPV use behavior to providers when also given the chance to discuss their IPV victimization experiences. Similarly, providers expressed a preference for asking about IPV use and experiences simultaneously in order to ‘get the full picture.’ Concurrent inquiry regarding IPV use and IPV experience is an important avenue for clinical research, especially given male Veterans’ reports of IPV experiences [24, 42] and female Veterans’ reports of IPV use [19, 43].

The present study also highlights the difficulty reaching consensus regarding the setting in which IPV use screening should take place. Many respondents (both patients and providers) believed that mental health clinicians may be better prepared to screen for IPV. However, most responders also agreed that primary care, due to its broader reach, would be an appropriate setting for IPV use screening implementation. Certainly, a primary care setting sees more patients and thus would be able to screen a larger number of people. However, primary care providers also often report being overburdened by screening, having a large volume of patients, being under-staffed, and not have enough time with each patient to conduct the many screenings that are already currently required [44–48]. Future research should formally evaluate the feasibility and effectiveness of screening for IPV use in these settings.

### Limitations

While generating important insights, this study has several limitations. First, study findings may not be transferable to other healthcare settings, including other VHA medical centers. Despite our relatively small patient sample size, we achieved data saturation across both patient and provider samples. However, consensus was not reached regarding preferences for the setting(s) in which IPV use screening should be implemented among either patient and provider participants; it is possible that additional participants would have clarified preferences for IPV screening setting(s). Second, most of the Veterans interviewed were men who served in the military over 20 years ago. As such, the present study may reflect generational and cohort effects. This type of research should be conducted with younger Veterans, including those who have served in the recent conflicts in Iraq and Afghanistan as IPV is a particularly salient issue for this population (e.g., [49]). Future studies should also seek to include more women, as well as assess how socio-demographic factors may impact perspectives and preferences for IPV use screening. Finally, we did not base patient recruitment on prior history of IPV events (use or experience) nor stratify our analysis based on this group membership. Perspectives regarding IPV screening may differ based on a person’s history of IPV events, an important consideration for future research.

## Conclusions

This study provides insights from patients and providers that can help to inform the development and implementation of IPV use detection and care practices in healthcare settings. Study findings discuss the benefits and challenges of implementing IPV use screening within VHA, the development of clinical guidelines for IPV use inquiry and follow up procedures, training protocols for IPV use screening, additional treatment options for IPV use, and a comprehensive IPV detection tool that screens for IPV use and experiences concurrently. Screening for IPV use requires methods that are accurate, easy to administer, and acceptable to patients and providers. Our findings extend prior research on provider and patient perspectives on screening for IPV experiences and are especially timely for VHA. The VHA is the largest integrated healthcare system in the US, and as such, it has the potential to develop, implement, and disseminate acceptable practices for comprehensively identifying and addressing IPV as well as serve as a model of care for other healthcare settings.

## Supplementary information

**Additional file 1.**

## Data Availability

To maintain confidentiality of individuals interviewed, the qualitative dataset generated and analyzed during this study is not publicly available. The sensitive nature of the research study and narrative statements of participants preclude requesting participants to consent and provide HIPAA authorization for disclosing the qualitative data.

## Abbreviations

US: United States
IPV: Intimate partner violence
VHA: Veterans Health Administration
PTSD: Posttraumatic stress disorder
FGD: Focus group discussions
MD PCP: Physician/primary care providers
