# Supplementary material for: Patient and provider barriers, facilitators, and implementation preferences of intimate partner violence perpetration screening
Source: BMC Health Serv Res. 2020 Aug 13;20:746. doi: 10.1186/s12913-020-05595-7 (PMC7424651; doi:10.1186/s12913-020-05595-7)
Supplement: Supplementary file 1 — Additional file 1. [file 12913_2020_5595_MOESM1_ESM.docx]

# Additional File 1

# Interview Guide (Veteran Participants)

1. During your appointments at the VA, has a provider ever asked you about your relationship? What about whether you or your partner have ever been aggression or violent towards each other? (a partner can be a girlfriend, boyfriend, spouse, sexual partner, etc.)

*If Yes 🡪*

1. In what type of appointment were these questions asked?
2. What type of provider asked these questions? (MD? MH? RN? Other?)
3. When you were asked questions, what did you experience? How did you feel about being asked those questions?
4. What was it like for you to respond?
   1. Probe, if needed: to what extent were you offended by such questions, comfortable answering these types of questions, answered the questions honestly, etc.
5. What did you like about how the provider asked you about this topic?
6. What did you wish was different?
7. How would you feel or react if a VA healthcare provider asked you about your violence or aggression towards a partner?
8. During a VA appointment, if a provider asked you about whether you use violence or aggression towards your partner:
   1. How offended would you feel by such questions?
   2. How comfortable would you be answering these types of questions?
   3. How likely would you be to answer the questions honestly?
      1. What would make it difficult to answer honestly?
9. What would make it easier to talk with your healthcare provider about these topics?
   1. What types of health care providers would feel more comfortable discussing these topics?
   2. What else would make it easier for you to respond honestly to providers asking about violence or aggression towards a partner?
10. What might get in the way of talking with your healthcare provider about these topics?
11. During a VA appointment, if a provider were to ask you questions about your anger, violence, or aggression directed towards a partner, *how* would you want them to ask you these questions?
    1. If unclear, prompt: what would your preference be between a self-report on a questionnaire while you wait for your appointment, directly in the appointment, other methods?
12. During a VA appointment, if a provider were to ask you questions about your anger, violence, or aggression directed towards a partner, *where* (in what setting) would you want them to ask you these questions?
    1. If unclear, prompt: would you prefer screening occur in primary care clinics, mental health clinics, other clinics, elsewhere?

**Interview Guide (Provider Participants)**

1. First, I’d like to start with your past experiences and current practices. Have you ever asked patients whether they use violence or aggression towards a relationship partner (such as physical violence, sexual violence, psychological or emotional violence, or stalking)?
   1. What are your reasons for asking or not asking?
   2. In what circumstances do you ask?
   3. How routinely do you ask?
   4. Whom do you ask?
   5. How do you usually ask?
   6. What is your sense of how your patients feel about being asked?
   7. What do you do if the patient discloses using violence?
2. What gets in the way of asking patients about their use of violence and aggression in relationships?
   1. What do you think is needed to change this?
3. How prepared or comfortable do you feel screening for intimate partner violence use with your patients?
   1. What would you need to feel more prepared or confident to routinely screen your patients for intimate partner violence perpetration?
4. What do you think would be the best way to screen for IPV perpetration in relationships?
   1. If needed, prompt: should it be face-to-face, paper screening, computer screening, integrated into routine care, prior to encounter, etc.?
5. Whose role do you think it should be to screen for perpetration of violence in relationships?
   1. If IPV perpetration screening were to be implemented in VHA, *where* (in what settings/clinics) do you think it should be implemented?
